# Motor-Sensory Learning in Children with Disabilities: Does Piano Practice Help?

**DOI:** 10.3390/children12030335

**Published:** 2025-03-07

**Authors:** Simon Strübbe, Susmita Roy, Irina Sidorenko, Renée Lampe

**Affiliations:** 1 Research Unit of the Buhl-Strohmaier Foundation for Cerebral Palsy and Pediatric Neuroorthopaedics, Department of Orthopaedics and Sports Orthopaedics, School of Medicine and Health, TUM University Hospital Rechts der Isar, Technical University of Munich, Ismaninger Str. 22, 81675 Munich, Germany; simon.struebbe@tum.de (S.S.); susmita.roy@tum.de (S.R.); irina.sidorenko@tum.de (I.S.); 2 Markus Würth Professorship, Technical University of Munich, Ismaninger Strasse 22, 81675 Munich, Germany

**Keywords:** motor-sensory disabilities, cerebral palsy, spasticity, instrumental therapy, developmental music therapy

## Abstract

**Background/Objectives:** Patients with physical disabilities, like cerebral palsy, the most common movement disorder in childhood, can benefit from instrumental therapy using piano. Playing the piano promotes the interaction between different brain regions and integrates motor skills, sensory skills, musical hearing, and emotions. A pilot music study examined the effects of six months of piano lessons on hand motor skills and musical hearing in groups of children with motor disabilities. **Methods:** The allocation to the group was not randomized. Various tests, including the standardized Box and Block Test (BBT) and piano tests, assessed hand motor skills. Musical hearing was evaluated, and a questionnaire was used to determine the participants’ enjoyment and experience with the piano lessons. The regularity, tempo of keystrokes, and synchronization between the two hands were assessed and compared to evaluate the effects of six months of piano training. **Results:** After six months of piano training, statistically significant improvements were observed in the BBT, as well as in the regularity and tempo of the non-dominant hand. The children showed significant improvement in hand-motor control, moving 27.3% more cubes in the BBT. Regularity and tempo in piano playing, especially in the non-dominant hand, also improved. Moreover, 55% of the children better recognized the correct pitches of notes. **Conclusions:** Thus, this study supports the concept that piano lessons are an effective form of physical therapy for the development of hand motor skills and musical hearing.

## 1. Introduction

Music is one of the oldest and most natural human activities, requiring no single region of the brain, but rather the interaction and coordination of several areas of the brain. These include the premotor cortex, which is involved in the formation of movement patterns; the auditory cortex, which is responsible for hearing; and the prefrontal cortex, which is associated with perception, memory, emotions [1]. Studies clearly show that motor timing functions, such as hand coordination, are not controlled by a single region of the brain [2]. Instead, they are performed by a network of regions that control specific movement parameters. Furthermore, eye-hand coordination is predominantly regulated by the cerebellum [3]. In contrast to speech, music is distinguished by a rhythmically structured and hierarchically complex metrical organization. This structure activates the motor regions of the brain, even during passive listening, a phenomenon that has been leveraged in therapeutic contexts for individuals with stroke or Parkinson’s disease to enhance gait patterns through rhythmic auditory stimulation [4,5]. The tendency to spontaneously tap in synchrony with a musical beat, emerges naturally, even in individuals without formal musical training [6]. The activation of motor regions during music listening is notably more pronounced if the individual has previously performed the piece of music [7]. This co-activation of motor and auditory regions operates bi-directionally: the auditory cortex is active during music performance, even in the absence of auditory feedback (e.g., when the sound is muted) [8].

The aforementioned complexity renders music-based approaches a particularly efficacious therapeutic instrument for patients afflicted with motor disorders with and without comorbidity, like cerebral palsy (CP). Our intervention group is heterogeneous; however, CP comprises a large group of several motor disorders [9], which is caused by a non-progressive damage of the developing brain, that occurs before, during, or shortly after birth [10]. Motor disabilities are often accompanied by speech development disorders, spatial and positional perception disorders, learning disabilities, epilepsy, spasticity (affecting up to 80% of individuals), dyskinesia, or ataxia [11]. All of them may benefit from neurorehabilitative music training.

In addition to conventional treatments such as physical therapy, occupational therapy, and speech therapy, music-based approaches are now gaining attention as treatment [12,13,14]. Music-based therapy can be conducted individually or in groups, either actively (e.g., playing instruments) or passively (e.g., listening or composing music). These approaches include techniques such as rhythmic auditory stimulation, therapeutic instrumental music performance, and patterned sensory enhancement and have been shown to improve gross and fine motor skills, influence gait, and promote balance in neurological patients [14,15,16,17]. In addition to motor benefits, music therapy reduces stress, regulates emotions, and promotes cognitive and communication skills [18,19].

The majority of studies employed the piano or keyboard as the primary musical instrument for therapeutic musical performance [20]. In contrast to instruments such as the violin, the piano does not require holding or tone shaping, allowing patients to play, even with functional restrictions in their hands, while seated in adapted wheelchairs. Immediate feedback from playing challenging key sequences helps focus on performance and listening. The repetitive finger movements, coordination of hands, hearing, and memory likely promote motor skill development and brain activation [2,21], supporting communication between brain regions.

Given the piano’s proven therapeutic impact, the present pilot study aimed to explore the effect of regular piano training on children with motor disorders. The study aimed to assess whether regular piano lessons can improve the hand-motor functional skills, coordination, and musical perception of the participants through motor-sensory learning. Furthermore, the study was conducted not only for medical and research purposes but also to inspire a lasting interest in music, enriching the children’s daily lives and overall well-being. Through questionnaires, participants’ experiences were examined to gain deeper insights into the learning process. Positive results from the study provide further evidence of the favorable effect of instrumental therapy as a new form of neurorehabilitative therapy.

## 2. Method

This study was approved by the internal ethics committee of the Faculty of Medicine of the Technical University of Munich with the number 2020-7_2-S-SB, prior to its commencement. Recruitment for this pilot study began in early November 2023 and concluded in mid-December 2023. Written informed consent was obtained from the parents of all participants before the project started.

**Figure 1:**
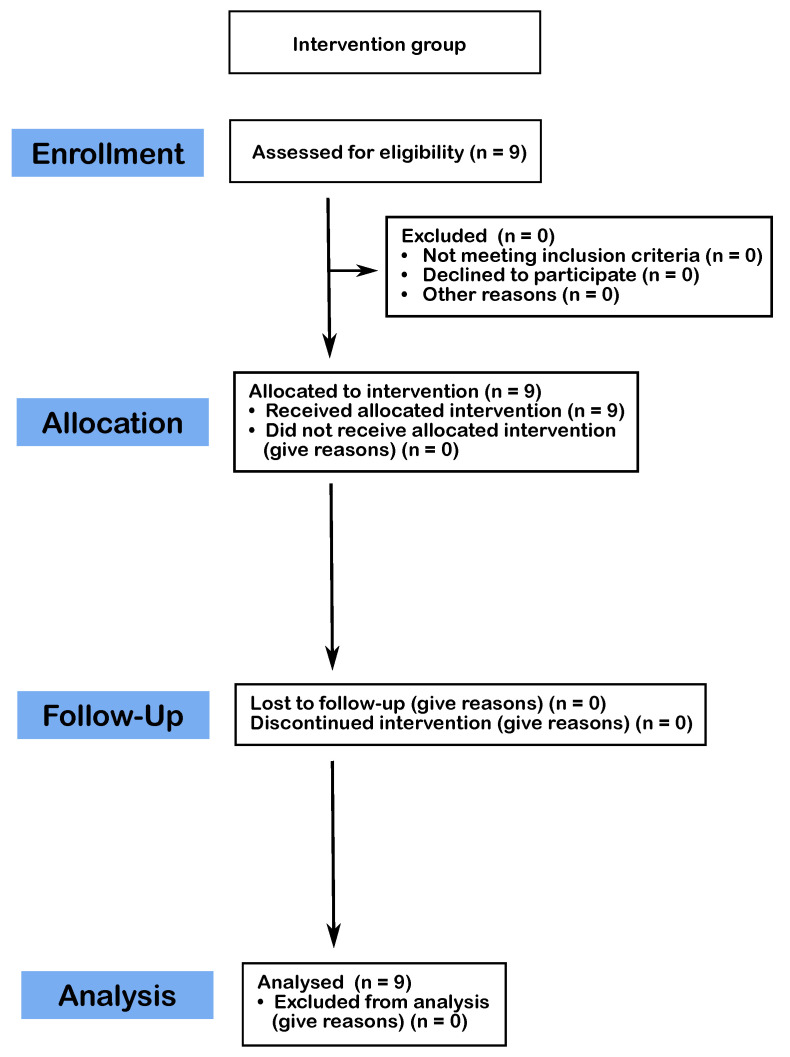
CONSORT 2010 flow diagram modified for a non-randomized study.

The study was specifically designed to accommodate a mixed-methods research approach, integrating both qualitative and quantitative methodologies. The study for this intervention (piano training) is registered at Deutsches Register Klinischer Studien (DRKS). Additionally, The CONSORT 2010 flow diagram, which has been modified for a non-randomized study, is shown in Figure 1.

Two professional piano instructors participated in the piano training. One instructor was responsible for the students enrolled in the school for physically disabled children, while the other trained the child attending kindergarten. Both instructors have experience teaching children with disabilities. The piece selected for the lesson was Mozart’s Piano Concerto No. 20 in D minor KV 466.

### 2.1. Participants

Participation in the study was voluntary. Nine children (eight male and one female) with physical disabilities participated in this study. The participating children ranged from 6 to 16 years of age and presented with different neuroorthopedic diagnoses. A common characteristic among the participants is that eight were recruited from a specialized school for physically disabled children. This institution cares for children with special educational needs, with an emphasis on fostering physical and motor development.

Additionally, one child with a neuro-orthopedic condition age to the others was recruited from a regular clinical check-up. This child was about to start school and was attending a regular kindergarten when the study began. The requirement for the participants was identical: none of them had received piano lessons from a professional teacher prior to the study. The only difference in training conditions was the location—eight children trained at their school with the school’s piano teacher, while the other child trained with a private piano teacher, and measurement took place at the teacher’s home.

The degree of mobility of the children was classified according to the Gross Motor Function Classification System (GMFCS) [22]. The GMFCS scale ranges from I to V, with level V corresponding to the highest degree of immobility. The GMFCS levels of the children in this study varied from I to III, and the classification was made by a senior orthopedic specialist. Table 1 shows the participants’ information in addition to their GMFCS level.

All participating children formed a piano orchestra that later accompanied the professional pianist during the piano concert. The notes were rewritten in six different voices to accommodate each child’s ability.

### 2.2. Design

**Figure 2:**
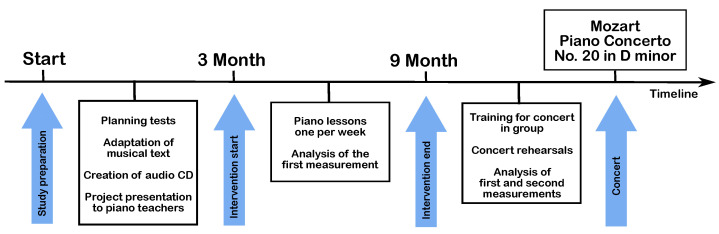
The stages of the study from planning to the final concert.

The steps of the study, from planning to the final concert, are illustrated in Figure 2. The initial measurements, described in detail in Section 2.3, were conducted before the start of the regular music lessons, and at this point, the children had not received any music training. After these initial measurements, the nine children received individual (not in group) piano instruction for 30 min once a week over six months, from professional piano teachers. After six months of lessons, a second set of measurements was performed. The second set of measurements was followed by a questionnaire with 12 questions concerning music education topics. In the final stage, the children‘s orchestra performed the concert together with the professional pianists and conductor in front of an audience of around 1000 guests.

### 2.3. Procedure

All tests and their evaluation for the participants were carried out before and after 6 months of piano training.

#### 2.3.1. Box and Block Test

The BBT is a standardized test that is often used to assess the gross motor skills of the hands [23]. The participants have one minute to move as many blocks as possible over a dividing wall inside the box. The performance of each hand is assessed by the number of blocks transferred. This number reflects finger dexterity (when picking up the block), finger strength (when holding the block), and hand quickness (when moving the block over the dividing wall). The hand must cross the dividing wall, and then the block can be dropped. If the number of blocks transferred is significantly below the normal value, this indicates a dysfunction of gross dexterity. The test focuses exclusively on arm and hand function. It is the most frequently used test to monitor the course of therapy in neurological rehabilitation measures.

#### 2.3.2. Tone Recognition Test

A test was developed to evaluate musical hearing. The test began by playing a sequence of four notes (C, D, E, and F) to the participant. Then, the participants were asked to close their eyes and listen to the fifth note, which was one of the four tones, and identify it. The test consisted of five rounds for each participant, in each of which one note had to be identified.

#### 2.3.3. Hand Motor Tests on the Piano (Piano Tests)

The 88-key E MIDI piano was used for the piano tests, the six months of piano training, and the final concert. During the test, the MIDI piano was connected to a computer, and the precise timing, strength of keystrokes, and the pressed piano keys were recorded with the Anvil Studio software (version 2023.02.01 64-bit). Six particular piano tasks were designed to evaluate fine finger motor function changes.

The participants were instructed to play five keys upwards and downwards, starting from a particular key, following the order of the tasks described below.

- **Task 1:** The five fingers of the right hand were placed on the piano keys C to G with the thumb on C, the index finger on D, and so on. The participants were instructed to press a single key of the piano with each finger three times (hereafter mentioned as triple key test), in ascending and descending order, starting from the thumb and returning to the thumb via all fingers. Data were recorded for three trials.
- **Task 2:** The same as task 1 but performed with the left hand.
- **Task 3:** The same as task 1 but performed with both hands simultaneously.
- **Task 4:** The five fingers of the right hand were placed on the piano keys C to G with the thumb on C, the index finger on D, and so on. The ascending and descending order of playing from C to G, pressing each key once (hereafter mentioned as a single key test), was recorded for three trials.
- **Task 5:** The same as task 4 but performed with the left hand.
- **Task 6:** The same as task 4 but performed with both hands simultaneously.

For each child, the mean time interval between consecutive keystrokes (stroke duration) and its standard deviation (irregularity) were calculated separately for the right and left hand. Irregularity reflects the variability of successive keystrokes and measures the uniformity of finger movements. In task 6, similar to task 3, the mean interval between keystrokes of the same two fingers in both hands was computed and defined as synchronicity. Short pauses disrupting the playing flow (e.g., due to re-instruction) were manually filtered out.

To evaluate the impact of piano training on hand motor action, the Wilcoxon signed-rank test was performed to metrics from task 1–6. This non-parametric test summed up the ranks of positive and negative changes in the metrics before and after training, with a *p* value of 0.05 as the significant threshold. The MIDI-Piano data were recorded using Anvil Studio software and were analyzed with MATLAB R2024a.

### 2.4. Involved Brain Regions in the Tests

**Figure 3:**
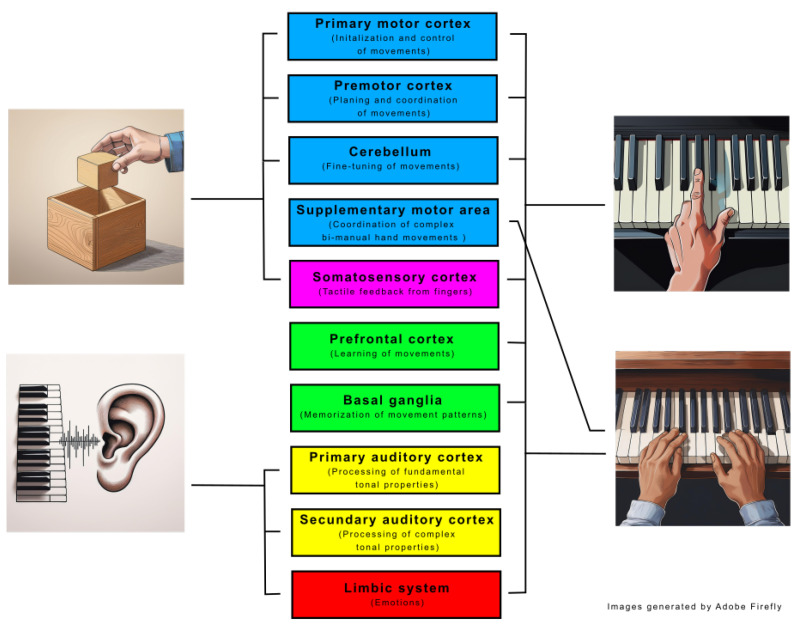
Primary brain regions involved in the BBT, single-hand and both-hand piano tests, and the hearing test, categorized by functional roles and represented with color coding: motor control (blue), tactile feedback (purple), learning (green), listening (yellow), and emotional processing (red).

Figure 3 illustrates the primary brain regions involved in the various tests conducted. The Box and Block Test primarily engages motor control regions (blue) and sensory feedback regions (purple). In contrast, the piano tests activate areas associated with learning and memorization (green), which are involved during the six months of piano training. Playing with both hands represents a more complex activity, engaging supplementary motor areas beyond those involved in simpler tasks. Meanwhile, auditory processing, as assessed in the hearing test, predominantly involves the auditory cortex (yellow). Furthermore, both listening to and performing music stimulate emotional processing regions within the limbic system (red).

### 2.5. Questionnaire for Participants

The entire study was evaluated using a questionnaire with 12 questions to obtain information directly from the participants about the piano lessons and conducted tests. The questions and the answers are presented in detail in Section 3.3.4.

## 3. Results

The detailed flow of participants through each stage of the study is described in Figure 1. The analysis included pre- and post-piano training tests from nine children. All the children participated in both the initial and the follow-up tests, which took place after six months of piano training. Table 2 provides an overview of the advances in hand-motor control observed among trained participants, as measured by the BBT and the piano tests.

### 3.1. Results of BBT

**Figure 4:**
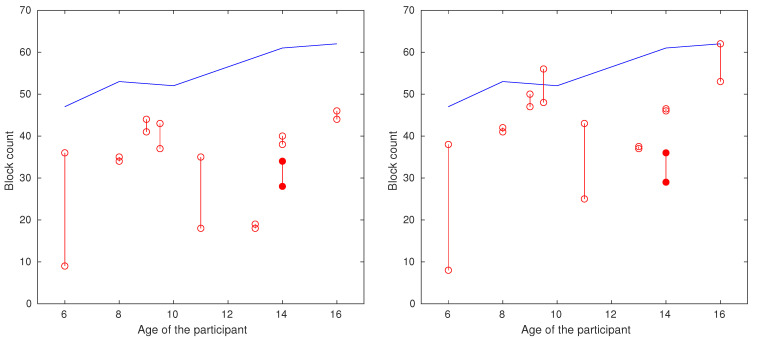
Results of the BBT with respect to participant age before (**left plot**) and after (**right plot**) six months of piano lessons. The red circles and the vertical lines connecting them illustrate the outcome for individual participants’ right and left hands (blank for males, filled for females). The horizontal blue line shows the mean value for healthy individuals of the corresponding age, taken from the literature [23].

The results of the BBT before and after six months of piano lessons are shown in Figure 4. The x axis (horizontal) and y axis (vertical) show the participant’s age and the number of blocks transferred. The red circles and the vertical lines connecting them illustrate the outcome for individual participants’ right and left hands (blank for males, filled for females). The horizontal blue line shows the mean value for healthy individuals of the corresponding age, taken from the literature [23].

**Figure 5:**
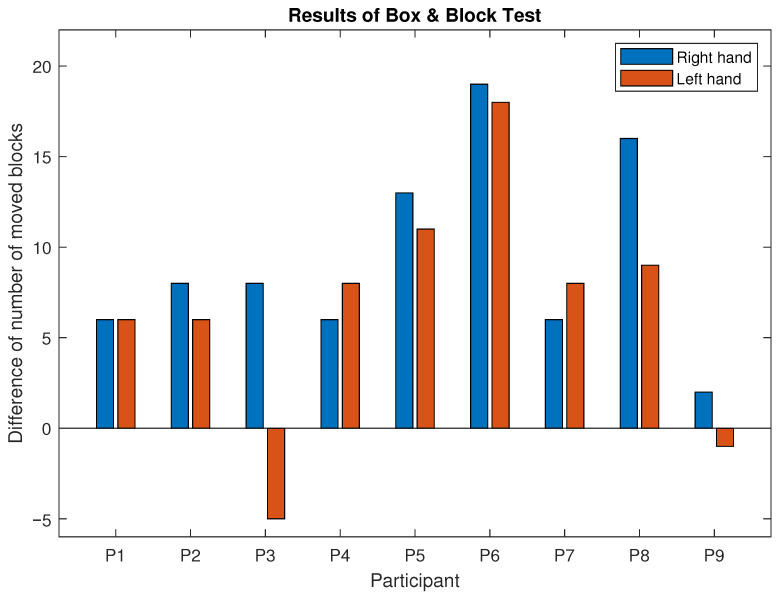
The difference in the number of blocks moved before and after six months of piano lessons. Red bars represent the results of the left hand, and blue bars represent the results of the right hand.

Figure 5 shows the difference in the number of blocks moved before and after training. Almost all participants a significantly improved performance in this test (*p*-value < 0.001). Median results showed that participants moved eight more blocks with each hand during the second measurement, which is an overall improvement of 27.3%, whilst an improvement of 29.9% is attributed to the dominant hand, and an improvement of 24.6% is attributed to the non-dominant hand. For the BBT, improvements for each participant are represented by positive values in Table 2.

### 3.2. Results of Tone Recognition Test

**Figure 6:**
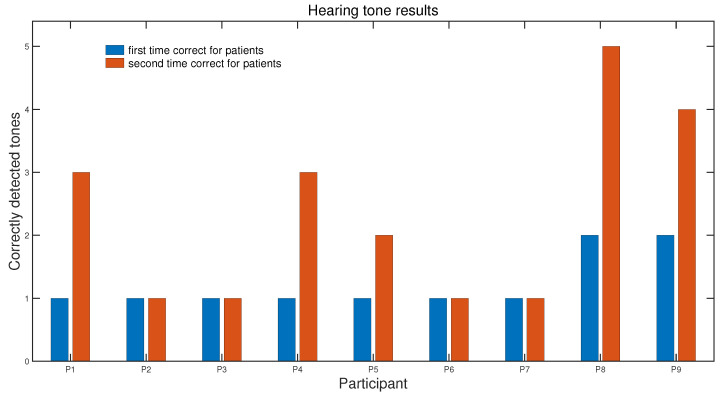
Results of the tone recognition test for children before (blue bars) and after (red bars) the piano lessons.

Figure 6 shows the results of the tone recognition test for the children before and after the piano lessons (blue and orange bars, respectively). The two bars corresponding to each child show the number of correctly recognized tones measured before and after training. The outcome shows only a moderate improvement in tone recognition: 55% of the participants noticeably improved their tone recognition in the second test, while the other 45% remained at the same level.

### 3.3. Results of the Piano Tests

#### 3.3.1. Visual Representation of the Ideal Piano Test

**Figure 7:**
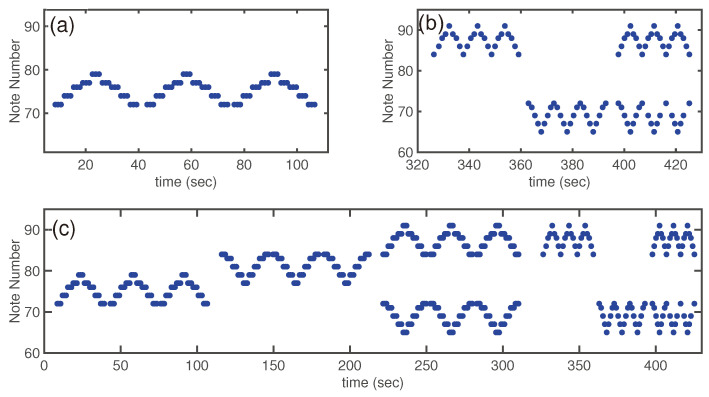
The ideal performance of the piano tests: (**a**) task 1; (**b**) tasks 4–6; and (**c**) the whole sequence of tasks 1–6.

To illustrate the ideal pattern of the piano test (Figure 7), all tasks described in Section 2.3.3 were performed by a healthy person, as described in [24]. Figure 7a shows the results recorded for task 1, Figure 7b shows the recordings for task 4 through task 6, and Figure 7c shows the results for a whole session (tasks 1–6). The regular pattern of playing, going up and down the keys, is quite regular and very symmetric.

#### 3.3.2. Visual Representation of the Piano Tests of a Participant

**Figure 8:**
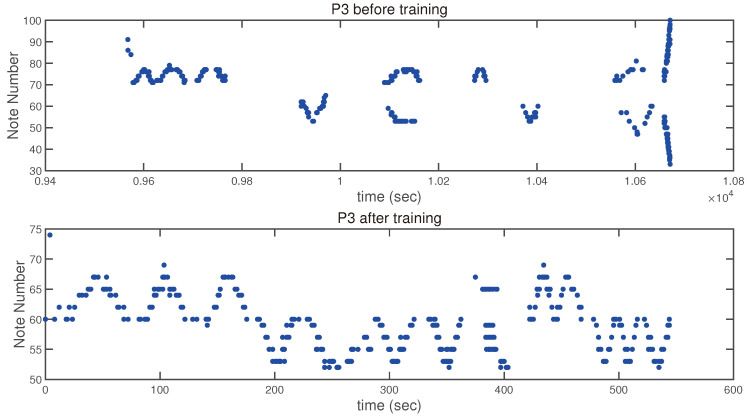
The results of the piano tests of a participant. The upper panel shows the results before and the lower panel shows the results after six months of piano training.

Figure 8 shows the results of the piano tests of a participant. The upper panel of Figure 8 shows the recorded tasks (1 to 6) for participant 3 before training. The participant plays very irregularly, and the recorded pattern shows significant time gaps during playing and an incomplete execution of the tasks. The bottom panel of Figure 8 (recorded after six months of piano lessons) shows a clear improvement in the performance of the same tasks. After six months of piano lessons, the uniformity during note playing improved significantly. The pattern became more symmetrical and regular. The time interval between individual presses approaches a constant value.

#### 3.3.3. Evaluation of the Piano Tests of All Participants

**Figure 9:**
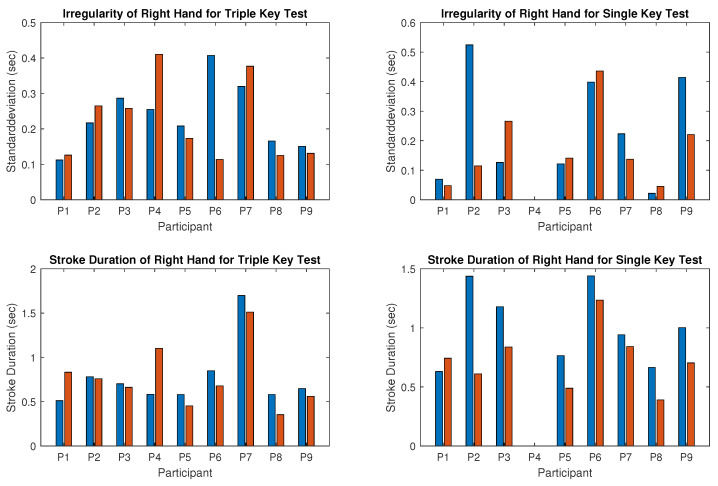
Irregularity (**upper panel**) and stroke duration (**lower panel**) for the triple key test (**left column**) and single key test (**right column**) for the right hand. Blue bars show the values before piano training and red bars show the values after six months of piano training.

**Figure 10:**
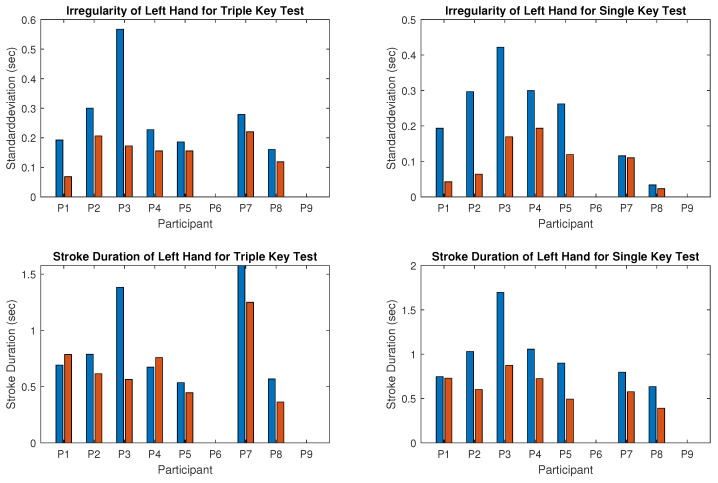
Irregularity (**upper panel**) and stroke duration (**lower panel**) for the triple key (**left column**) and single key (**right column**) tests for the left hand. Blue bars show the values before piano training, and red bars show the values after six months of piano training.

**Figure 11:**
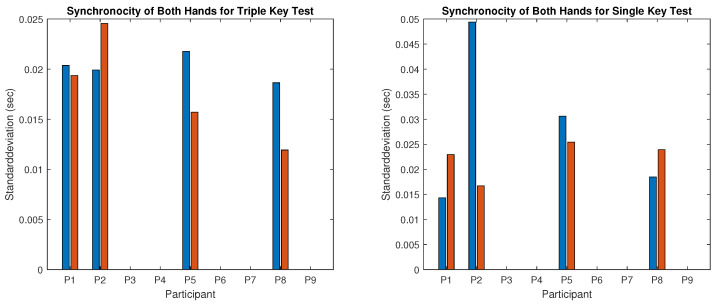
The synchronicity of both hands for the triple (**left**) and single (**right**) key tests before (blue bars) and after (red bars) 6 months of piano training.

The recorded MIDI files from nine participants were individually analyzed. Stroke duration and irregularity for the triple and single key tests were calculated separately for the right and left hands and summarized in Figure 9 and Figure 10, where blue bars represent values recorded before and red bars represent values recorded after the piano training. Synchronicity of stroke times for both hands in tests 3 and 6 is shown in Figure 11 (left: triple key test; right: single key test). Piano tests improvements are represented by negative values in Table 2, which also presents the mean performance gain between triple- and single-note playing. Details of the results are described below.

- Results for the right hand:
  The computed irregularity and stroke duration for the right hand, which was the dominant hand in seven of the nine participants, are illustrated in Figure 9. Reduced irregularity and shorter stroke duration indicate better performance. Regularity (opposite to irregularity shown in the upper panel of Figure 9) of the right hand improved in five of the nine participants during the triple key tests (Figure 9 left) and in four of the eight participants during the single key test (Figure 9 right). Participant 4 was unable to perform the single key test. Some participants showed no improvement, with either stable scores or slight deterioration. Statistical tests for right-hand regularity showed no significant changes (*p*-value > 0.05).
  The tempo (inverse of stroke duration shown in the lower panel of Figure 9) of the right hand increased in all participants except participant 1, who played slightly slower in both tests after training. For the whole group, this improvement was statistically significant (*p*-value < 0.05). Participant 4, with severe right-hand restriction, could only perform the triple key test and required multiple re-instructions, showing high irregularity.
- Results for the left hand:
  Figure 10 presents the results for the left hand, typically the non-dominant hand, showing clear improvements in all participants except 6 and 9, who could not perform the test due to motor limitations. Regularity (opposite to irregularity shown in the upper panel of Figure 10) increased significantly after the training. For instance, irregularity in the triple key test decreased by more than half for participants 1 and 3, and similar reductions were observed in the single key test for participants 1, 2, 3, and 5. Group results were statistically significant (*p*-value < 0.001). The tempo (inverse stroke duration, lower panel of Figure 10) improved significantly (*p*-value < 0.005) in both tests for most participants, except for participants 1 and 4, who played slightly slower in the triple key test.
- Correlation between improvement and dominant hand:
  For all participants except 4 and 6, the left hand (non-dominant) showed clear improvements in regularity and tempo. Participant 6, left-hand dominant, improved in the right hand during the triple key test, while the regularity in the single key test remained almost unchanged. Participant 4, also left-hand dominant, could not perform the right-hand single key test and showed no improvement in the triple key test despite multiple instructions.
- Results for the synchronicity of both hands:
  The averaged synchronicity of both hands in the triple and single key tests, measured as the standard deviation of keystroke offsets, is shown in Figure 11. Due to motor limitations, only participants 1, 2, 5, and 8 could perform this test. Figure 11 reveals no clear correlation between training and synchronicity improvement. Participants 5 and 8 improved in the triple key test, and participant 2 improved in the single key test, while others showed no change or reduced synchronicity. The *p*-value indicates no significant improvement.

#### 3.3.4. Results of the Questionnaire

The questionnaire and participants’ answers are summarized in Table 3. The main conclusion that can be drawn from analyzing the questionnaire is the positive attitude of the children towards music lessons and tests. Regardless of individual successes, all participants demonstrated a high level of emotional wellness during the piano lessons, tests, and the final concert.

## 4. Discussion

In this pilot study, Box and Block Test (BBT) and piano music tests were conducted on nine children and adolescents diagnosed with neuro-orthopedic conditions. The effects of six months of one-on-one regular piano lessons on hand coordination, gross motor skills, and musical hearing were examined, along with gaining new insights into the participants’ experiences of the piano learning process through questionnaires.

While the effects of music therapy on stroke patients have been widely studied in neurological studies [25,26,27], research on the rehabilitation effects of instrumental therapy in children with neurological conditions remains limited. Neuro-orthopedic diseases, such as cerebral palsy (CP), can lead to fine and gross motor impairments in the upper extremities, and hand function is often significantly affected for them [28].

The main results of the present study are outlined again to provide a brief and concise overview of the study.

- The results of the standard BBT show a significant improvement (*p*-value < 0.001) after six months of piano training in a group of children and adolescents with disabilities.
- The regularity and tempo of consecutive and repeated finger strokes by the left hand, typically the non-dominant hand, also improved significantly during piano tests after the training (*p*-value < 0.001 and *p*-value < 0.005, respectively).
- The improvement in the regularity of the right hand for similar tests was not significant, but the tempo of the right hand improved significantly (*p*-value < 0.05).
- The questionnaire responses indicate that all participants thoroughly enjoyed the piano lessons and expressed a strong appreciation for music.

In the present study, piano training led to a median increase of eight blocks in the BBT for each hand of the participants, indicating significant improvement, even after short-term piano training. The BBT is widely used as an outcome measure in studies evaluating various therapies such as constraint-induced movement therapy (CIMT) [29], robot-assisted therapy [30] etc., particularly for assessing improvements in hand motor function in stroke patients [31]. Compared to these therapies, the present results indicate a greater increase in the number of blocks moved, suggesting a potential advantage of piano training in enhancing hand motor function. Although the BBT is a direct and relatively simple outcome measure compared to other piano tests used in this study, the observed improvement aligns with Karatekin and Icagasioglu [32], who reported significant gains in block-moving performance in patients with CP after three months of weekly piano lessons. Playing the piano offers numerous benefits, including improved hand–eye coordination and dexterity. Regular piano practice may also improve everyday activities such as the complex functional skills of fingers, as the tactile perception of the fingers improve and hand–eye coordination is trained. This may be why the Box and Block Test improved after regular piano lessons. In a study conducted by Villeneuve et al., a small group of patients who had suffered a stroke received piano lessons after just 3 weeks, and were able to demonstrate improvements in hand dexterity and in the results of the Box and Block Test [25].

Better piano playing is associated with lower irregularity and consistent keystroke duration. Our piano playing tests revealed a difference in score between the dominant and non-dominant hand: while the dominant hand showed no clear improvement after training, the non-dominant hand significantly improved the performance in the single key and triple key tests. This improvement in the less-trained hand aligns with the expectation that regular practice benefits the hand less frequently used.

The reduction in irregularity, as measured by the standard deviation of keystrokes, indicates greater consistency in playing, aligning with the findings of Lampe et al. [33], who also observed increased keystroke uniformity due to regular piano training and finger exercises. Tempo improved for nearly all participants, consistent with Chong et al. [13]. While overall hand synchronization showed limited progress, two participants improved coordination in both hands after six months. Despite the short training period, clear improvements were observed, possibly due to the benefits of shorter intervals between test repetitions, which could help participants better recall and perform the task.

Nevertheless, this study has certain limitations that should be explored and addressed in subsequent research. While this study did not include a healthy cohort, which could be viewed as a limitation, this does not compromise the validity of our findings, because we compared the results of BBT with the reference values from the literature [23] and the improvement in the participants in the piano test with their initial untrained condition. Furthermore, the heterogeneity of the patient cohort could be another limitation. A homogeneous patient cohort would, for instance, allow for a more precise examination of relationships between clinical parameters, such as specific neurological disorders like CP, and hand motor improvement. Although the members of the group have different abilities and heterogeneity in terms of their individual needs, there is still a common feature, which is that they suffer from motor impairments that limit their functional abilities. However, the heterogeneity of the group can also be an advantage, as it can be said that people with deficits, regardless of diagnosis, can benefit from playing the piano.

Given the age group of the participants in this study, their neural system is still immature and benefits from plastic changes such as myelination, neurogenesis, and dendritic growth. In our study, we can assume that these aspects also played a role. Ongoing myelination contributes to an increase in white matter volume from childhood to late adolescence [34,35]. However, it is important to emphasize that we can only speculate about these neuroplastic effects in our study, as no imaging techniques such as fMRI or DTI were used to validate these hypotheses.

Furthermore, research has shown that long-term musical training and the development of sensorimotor skills can drive significant neuroplastic changes. These changes occur in both the developing and adult brain, influencing white and gray matter as well as cortical and subcortical structures [36,37]. Active musical engagement strengthens the connection between perception and action by integrating sensory, motor, and multimodal brain regions. Furthermore, music-based activities enhance rehabilitation and neurotherapy by making the process more engaging and enjoyable. By stimulating and linking different brain regions, music can help restore impaired neural connections and improve overall brain function [13,24,38]. Following the promising outcomes of our study, further research is needed to standardize protocols for piano therapy and deepen our understanding of its mechanisms. Furthermore, future research in this area could utilize a control group of a comparable sample size, using the same methods, to determine and compare the level of improvement or the effect of the intervention across the two groups (control and patient groups). The Reporting Guidelines for Music-Based Interventions provide a solid framework for advancing research in this field [32].

Despite the limitations of the present study, our findings provide evidence of improvements in motor-sensory learning and piano playing, as well as a positive attitude toward music. Based on existing research, we suggest that these results could have a significant positive impact on the children’s daily lives and future.

## 5. Conclusions

Engagement in musical activities has been demonstrated to optimize the functional abilities of the hands. In particular, progress was demonstrated in the piano exercises by the non-dominant hand. Specific basic motor skills are required for the two hands to work together, which may be lacking due to spasticity. Nevertheless, it can also be assumed that, in this case, the hands are better perceived and manifested. Engaging in music enables a more sensitive perception of sounds, regardless of the disability.

It is also important to emphasize the emotional effect of our study. Despite the motor impairments, all the participants who took part in this study enjoyed the piano lessons and learned to play a piece of music composed of notes that were not originally related. This proved to be an extremely challenging task. The beautiful Mozart melody was only achieved in a group setting, where all the participants played together to form a piano orchestra. The final concert was particularly enjoyable for both the students and their parents. As revealed from the questionnaire, the joy of playing the piano is independent of any disability. The participants were proud of their progress, as evidenced by the questionnaire and the excellent results achieved in the performed tests.

## Figures and Tables

**Table 1:** Demographic characteristics of participants and the classifications of their mobility according to GMFCS and their hand performance in daily life.

Participant	Gender	Age (Years)	Dominant Hand	GMFCS
P1	M	9	Right	I
P2	M	8	Right	I
P3	F	14	Right	II
P4	M	11	Left	II
P5	M	9	Right	II
P6	M	13	Left	III
P7	M	14	Right	I
P8	M	16	Right	I
P9	M	6	Right	II

**Table 2:** Improvements in the trained participants for the B&B Test (positive values) and the piano tests (negative values).

Participant	BBT r. h.	BBT l. h.	Irr. r. h.	Dur. r. h.	Irr. l. h.	Dur. l. h.	Syn. b. Hands
P1	+13.6%	+14.6%	−9.7%	+40.3%	−71.2%	+5.6%	+27.6%
P2	+23.5%	+17.1%	−28.0%	−30.1%	−54.8%	−31.8%	−21.4%
P3	+28.6%	−14.7%	+50.3%	−17.3%	−64.7%	−53.8%	NAN
P4	+38.9%	+22.9%	NAN	NAN	−33.5%	−9.3%	NAN
P5	+28.3%	+29.7%	−0.3%	−29.0%	−35.5%	−30.9%	−22.4%
P6	+105.5%	+94.7%	−31.3%	−17.2%	NAN	NAN	NAN
P7	+15.0%	+21.1%	−10.4%	−10.8%	−13.0%	−24.2%	NAN
P8	+34.8%	+20.5%	+42.1%	−40.3%	−28.7%	−37.1%	−3.3%
P9	+5.6%	−11.1%	−30.0%	−21.7%	NAN	NAN	NAN

Abbreviations: BBT = Box and Block Test; h. = hand; r. = right; l. = left; b. = both; irr. = irregularity; dur. = duration; syn. = synchronicity; NAN = not a number.

**Table 3:** Questionnaire for patients.

Q. No.	Question	Answer	Percentage
1.	Do you like taking piano lessons?	Yes	100%
		Sometimes	0%
		No	0%
2.	Was it difficult to learn to play the piano?	Yes	31%
		A little bit	31%
		No	38%
3.	What do you particularly like about playing the piano?	Techniques	16%
		Melody	38%
		Both	46%
4.	Where are the high and low tones?	Correct	84%
		False	16%
5.	How do you learn to read the musical notes?	Colors	62%
		Numbers	15%
		Letters	15%
		Notes	8%
6.	Do you have a piano or keyboard at home?	Piano	38%
		Keyboard	24%
		No	38%
7.	Do you like playing for others?	Yes	77%
		Sometimes	15%
		Never tried	8%
8.	Do you enjoy playing with others?	Yes	77%
		Depends	15%
		No	8%
9.	Does anyone else in your family play the piano?	Yes	54%
		No	46%
10.	What do you feel after you learn to play the piano?	Proud	100%
		Satisfied	0%
		Disappointed	0%
11.	Were the piano tests boring or enjoyable?	Enjoyable	100%
		Tolerable	0%
		Boring	0%
12.	What is your favorite music?	Rock, Classical, Rap, Schlager, Everything except Schlager, Drum music, Children’s music, Robot music	

## Data Availability

The data that support the findings of this study are available from the corresponding author upon reasonable request due to data privacy.
